# Repaired coarctation of the aorta, persistent arterial hypertension and the selfish brain

**DOI:** 10.1186/s12968-019-0578-8

**Published:** 2019-11-07

**Authors:** Jonathan C. L. Rodrigues, Matthew F. R. Jaring, Melissa C. Werndle, Konstantina Mitrousi, Stephen M. Lyen, Angus K. Nightingale, Mark C. K. Hamilton, Stephanie L. Curtis, Nathan E. Manghat, Julian F. R. Paton, Emma C. Hart

**Affiliations:** 1 Department of Cardiovascular Magnetic Resonance, Bristol Cardiovascular Biomedical Research Unit, Bristol Heart Institute, University Hospitals Bristol NHS Foundation Trust, Bristol, UK; 2 School of Physiology, Pharmacology & Neuroscience, Faculty of Biomedical Science, University of Bristol, Bristol, UK; 3 Department of Radiology, Royal United Hospitals Bath NHS Foundation Trust, Bath, UK; 4 Department of Radiology, Bristol Royal Infirmary, University Bristol NHS Foundation Trust, Bristol, UK; 5 BHI CardioNomics Research Group, Clinical Research and Imaging Centre-Bristol, University of Bristol, Bristol, UK; 6 Adult Congenital Heart Disease Unit, Bristol Heart Institute, Bristol Royal Infirmary, University Hospitals Bristol NHS Foundation Trust, Upper Maudlin Street, Bristol, UK; 7 Department of Physiology, Faculty of Medical and Health Sciences, University of Auckland, Park Road, Grafton, Auckland, New Zealand

**Keywords:** Coarctation, Hypertension, Circle of Willis, Vertebral artery

## Abstract

**Background:**

It has been estimated that 20–30% of repaired aortic coarctation (CoA) patients develop hypertension, with significant cardiovascular morbidity and mortality. Vertebral artery hypoplasia (VAH) with an incomplete posterior circle of Willis (ipCoW; VAH + ipCoW) is associated with increased cerebrovascular resistance before the onset of increased sympathetic nerve activity in borderline hypertensive humans, suggesting brainstem hypoperfusion may evoke hypertension to maintain cerebral blood flow: the “selfish brain” hypothesis. We now assess the “selfish brain” in hypertension post-CoA repair.

**Methods:**

Time-of-flight cardiovascular magnetic resonance angiography from 127 repaired CoA patients (34 ± 14 years, 61% male, systolic blood pressure (SBP) 138 ± 19 mmHg, diastolic blood pressure (DBP) 76 ± 11 mmHg) was compared with 33 normotensive controls (42 ± 14 years, 48% male, SBP 124 ± 10 mmHg, DBP 76 ± 8 mmHg). VAH was defined as < 2 mm and ipCoW as hypoplasia of one or both posterior communicating arteries.

**Results:**

VAH + ipCoW was more prevalent in repaired CoA than controls (odds ratio: 5.8 [1.6–20.8], *p* = 0.007), after controlling for age, sex and body mass index (BMI). VAH + ipCoW was an independent predictor of hypertension (odds ratio: 2.5 [1.2–5.2], *p* = 0.017), after controlling for age, gender and BMI. Repaired CoA subjects with VAH + ipCoW were more likely to have difficult to treat hypertension (odds ratio: 3.3 [1.01–10.7], *p* = 0.049). Neither age at time of CoA repair nor any specific repair type were significant predictors of VAH + ipCoW in univariate regression analysis.

**Conclusions:**

VAH + ipCoW predicts arterial hypertension and difficult to treat hypertension in repaired CoA. It is unrelated to age at time of repair or repair type. CoA appears to be a marker of wider congenital cerebrovascular problems. Understanding the “selfish brain” in post-CoA repair may help guide management.

**Journal subject codes:**

High Blood Pressure; Hypertension; Magnetic Resonance Imaging (MRI); Cardiovascular Surgery; Cerebrovascular Malformations.

## Background

Coarctation of the aorta (CoA) occurs in approximately 4/10,000 live births, accounting for 4–6% of all congenital heart defects [1, 2]. CoA is associated with increased risk of multiple cardiovascular complications including coronary artery disease, aortic aneurysm formation and cerebrovascular disease [3–5]. Arterial hypertension occurs in approximately 30% following CoA repair [6, 7] and is a unifying risk factor. Upper body hypertension would be predicted if CoA repair was inadequate [8] or if subsequent growth of the arch or repaired segment was suboptimal [9]. However, hypertension is common even in the presence of a good repair [3, 5, 10] and associated with autonomic imbalance [10]. Age at the time of the original CoA repair has been shown to contribute to subsequent risk of hypertension [11]. However, a high prevalence of hypertension has been subsequently demonstrated in children aged 7–16 years who were treated for CoA at a median age of 0.2 years and without residual significant arch obstruction [7]. Consequently, the reason why hypertension is so common in repaired CoA remains enigmatic.

Recently, our group investigated the role of the “selfish brain” hypothesis in the development of hypertension in-vivo, in human [12]. Humans with hypertension had higher prevalence of vertebral artery hypoplasia (VAH) and incomplete posterior circle of Willis (ipCoW), which was coupled with elevated cerebrovascular resistance (CVR) and diminished cerebral blood flow. Importantly, CVR was increased prior to the development of arterial hypertension and elevated sympathetic nerve activity (SNA) in untreated borderline hypertensive subjects, suggesting that the cerebral hypoperfusion occurred prior to overt activation of the sympathetic nervous system.

We now investigate the role of the “selfish brain” in hypertension following CoA repair. The hypothesis was that VAH with ipCoW (VAH + ipCoW) would be more prevalent in the repaired CoA population developing arterial hypertension compared to normotensive controls and this would predict the development of hypertension after CoA repair.

## Methods

### Study population

**Fig. 1:**
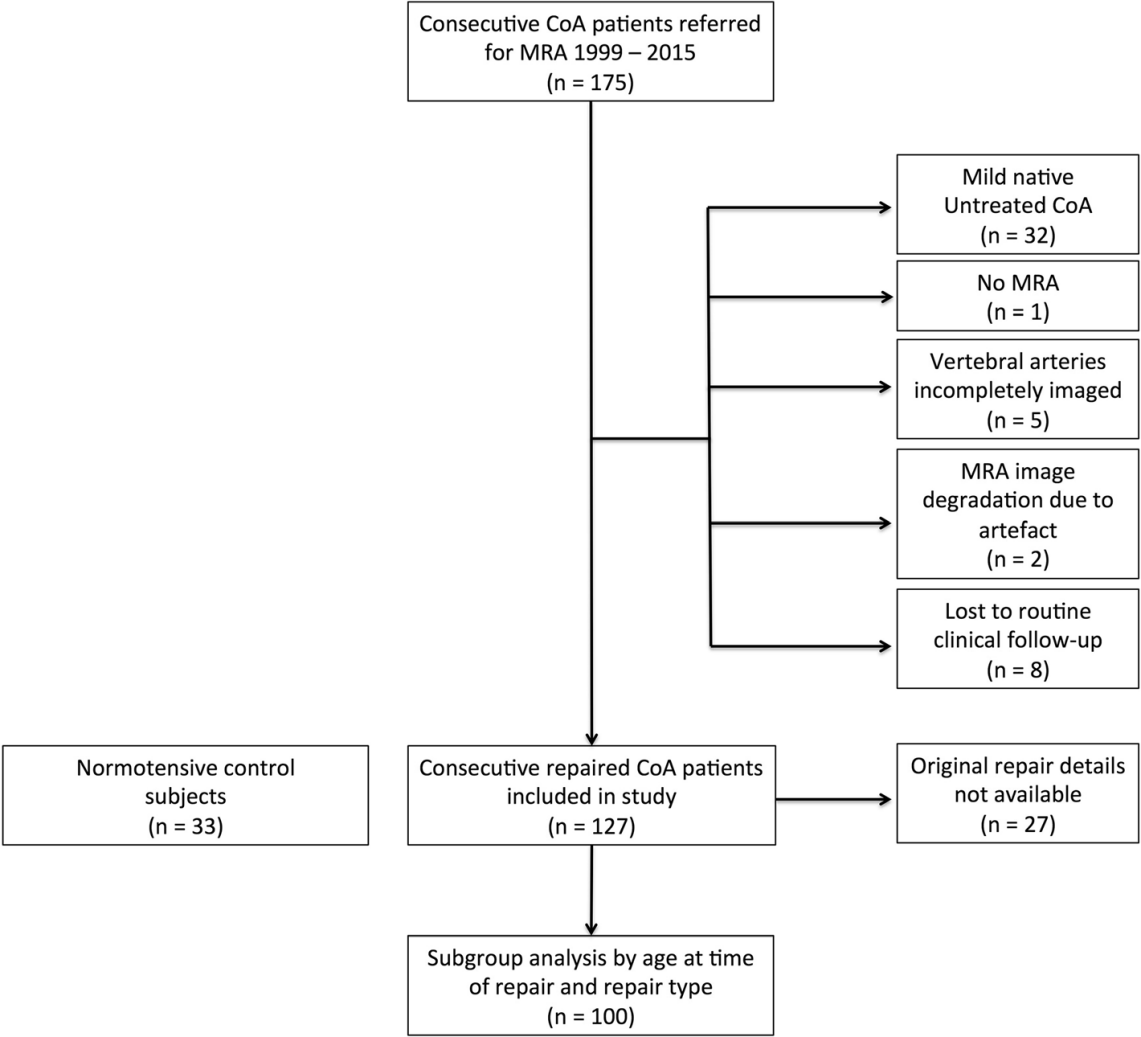
Flow chart demonstrating the study design. CoA = coarctation of the aorta, MRA = magnetic resonance angiography

The local Research Ethics committee confirmed that the study conformed to the governance arrangements for research ethics committees. A retrospective review of a prospectively maintained clinical database of consecutive patients with a history of CoA, > 16 years, undergoing routine clinical cardiovascular magnetic resonance (CMR) surveillance as part of their first presentation to the Adult Congenital Heart Disease Unit within the Bristol Heart Institute between 1999 and 2015 was performed. All patients provided written informed consent for their images to be used for research. Exclusion criteria included non-diagnostic intracranial time-of-flight magnetic resonance angiography (MRA) and subjects who did not undergo CoA repair or who were lost to follow-up (Fig. 1). Baseline demographic and clinical characteristics were recorded from electronic chart review including age and type of CoA repair, a documented diagnosis of hypertension and drug therapy. Where details of specific CoA repair type were missing or ambiguous (*n* = 27), e.g. where the repair was performed in an outside institution and the original operation note was not available, were excluded from subgroup analysis of the impact of repair type on VAH + ipCoW (Fig. 1). Average office systolic (SBP) and diastolic blood pressures (DBP) were acquired using an automated cuff (Omron Corporation, Kyoto, Japan), in accordance with International hypertension guidelines [13]. Uncontrolled hypertension was defined as office BP > 140/90 mmHg despite at least 2 anti-hypertensive medications [13]. Data from age and sex-matched normotensives, a subgroup from a prior research study [12], were used as a control group.

### MRA protocol

Aortic MRA had been performed in all repaired CoA subjects to assess for repair site complications. Briefly, following the injection of 0.1 mmol/kg of intravenous gadobutrol (Gadovist, Bayer Pharma AG, Berline, Germany), a 3D systemic arterial MRA at 1.5 T (Avanto, Siemens Healthineers, Erlangen, Germany) from thoracic apex to groins was acquired (TR/TE = 3.1/10.9 ms, flip angle = 25 degrees, voxel size = 1.1x1x1mm, matrix 448 × 265). Intracranial time of flight MRA is routinely performed at the time of first presentation imaging surveillance in all new subjects with history of repaired CoA presenting to our Adult Congenital Heart Disease Unit to screen for intracranial aneurysms as previously described [14]. In brief, a 3D time-of-flight MRA at 1.5 T (Avanto, SiemensHealthineers) with dedicated head coil to assess arterial anatomy (TR 38 ms, TE 5.28 ms, flip angle 25 degrees, voxel size 0.7 × 0.5 × 0.8 mm, field of view 200 mm, covering major arteries feeding into the circle of Willis. The normotensive controls were scanned using 3 T (GE HDx, General Electric Healthcare, Waukesha, Wisconsin, USA) to generate a 3D time-of-flight MRA (TR/TE 24/2.7 ms flip angle 20 degrees, voxel size 0.34 × 0.34 × 0.5 mm^3^, field of view 192x192x85 mm^3^).

### Aortic MRA analysis

Source aortic MRA data were routinely reported by a consultant cardiovascular radiologist and retrospectively independently reviewed by an imaging cardiologist with > 2 years’ experience blinded to clinical details, including CoA repair type, degree of residual narrowing and normotensive/hypertensive state. As previously described [15, 16], re-coarctation was defined when the diameter of the repaired CoA segment divided by the diameter of the descending thoracic aorta at the diaphragmatic hiatus was < 40%. Re-coarctation on imaging could not be directly assessed in stent repair due to artifact. However, there were no clinical features (such as arm-leg SBP discrepancy) to suggest a clinically relevant re-coarctation in any post-CoA repair subjects. Arch hypoplasia was assessed by the ratio of the minimum mid aortic arch diameter to the descending thoracic aorta at the level of the left atrium, as previously [17]. Multi-planar reformatted gadolinium-enhanced aortic MRA images were also reviewed for the presence of renal artery stenosis, defined as as > 50% focal reduction in vessel diameter [18].

### Intracranial arterial MRA analysis

**Fig. 2:**
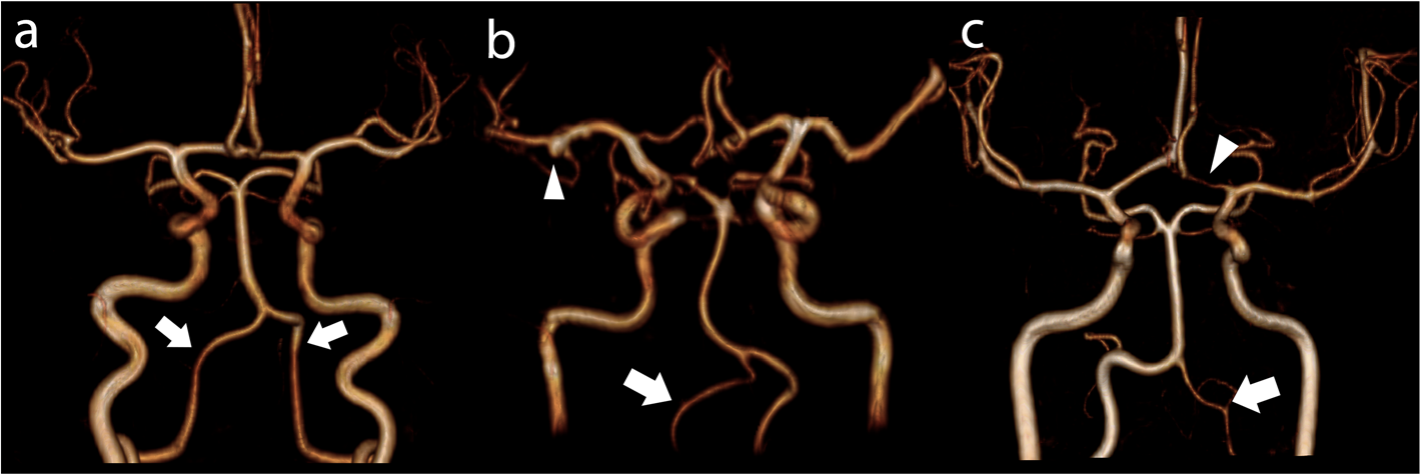
Vertebral artery hypoplasia. Panels a to c show 3D MRA reconstructions of the Circle of Willis and vertebral arteries. **a** Normal symmetrical vertebral arteries (arrows). **b** Right vertebral artery hypoplasia (arrow). 4 mm aneurysm of the distal right middle cerebral artery (arrowhead). **c** Left vertebral artery hypoplasia (arrow). Note incidental hypoplasia of the pre-communicating left anterior cerebral artery

**Fig. 3:**
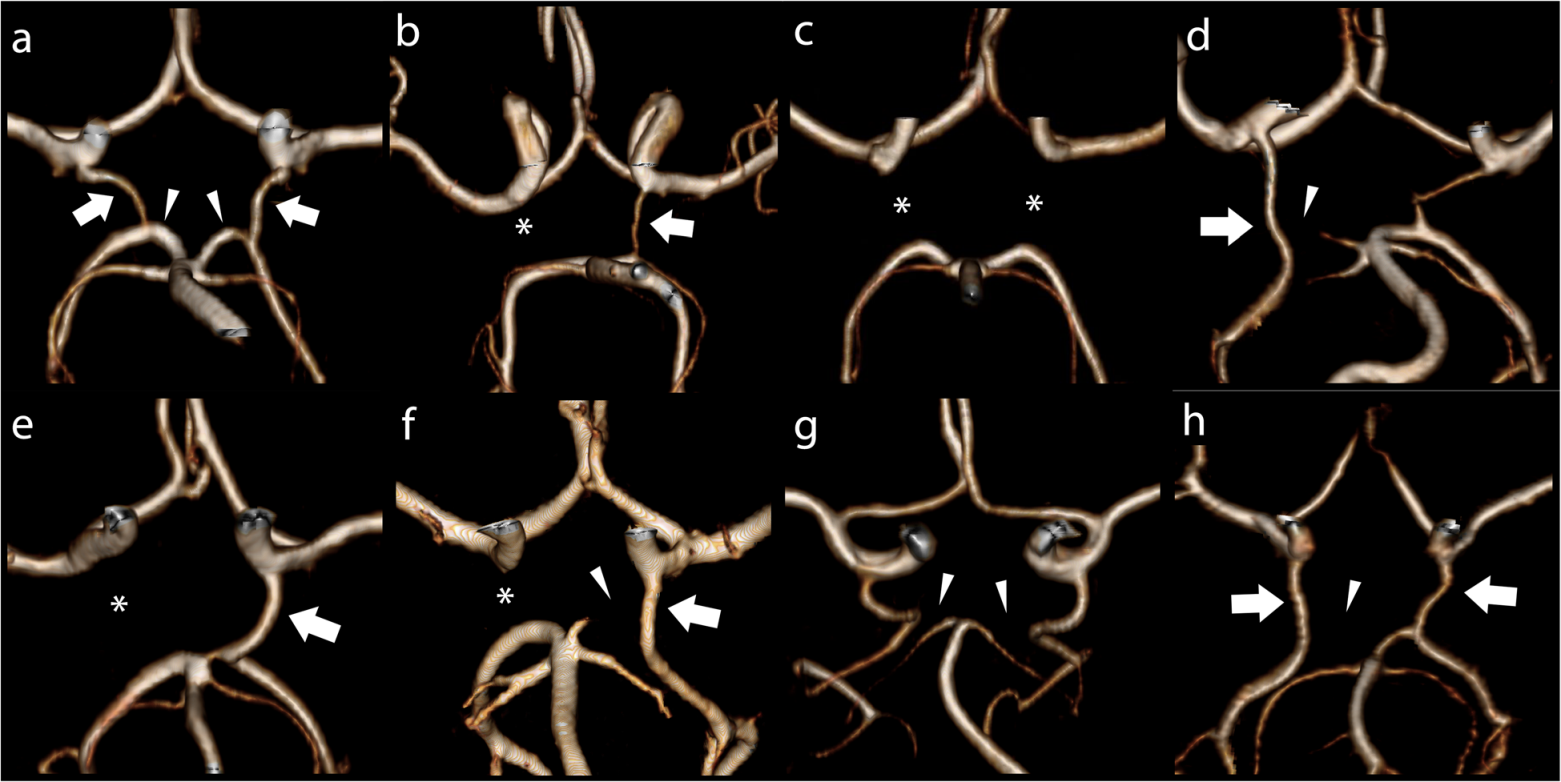
Variations of incomplete posterior Circle of Willis. Panels a to h show 3D MRA reconstructions of the Circle of Willis. PComm = posterior communicating artery; PCA = posterior cerebral artery. **a** Normal Circle of Willis. The PComms are indicated by arrows and precommunicating segment of the PCAs are marked by arrowheads. **b** Unilateral left PComm (arrow) and absent contralateral PComm (asterisk). **c** Bilateral absent PComms (asterisks). **d** Unilateral right foetal type PCA and severely hypoplastic ipsilateral precommunicating segment of the PCA (arrowhead). **e** Unilateral right foetal type PCA (arrow) and absent contralateral PComm (asterisk). **f** Unilateral left foetal type PCA (arrow), incomplete precommunicating segment of the left PCA (arrowhead) and absent right PComm (asterisk). **g** Bilateral foetal type PCAs with absent precommunicating segments of the posterior cerebral arteries (asterisks). **h** Bilateral foetal type PCAs (arrows) with absent precommunicating segment of right PCA (arrowhead)

Source MRA data were reviewed in 3 orthogonal multiplanar reformatted (MPR) planes with cross-referencing of images. Maximum intensity projection images were generated and reviewed. Scans were routinely reported by a consultant cardiovascular radiologist and retrospectively independently reviewed by a radiologist with > 3 years’ experience blinded to clinical details, including CoA repair type, degree of residual narrowing and normotensive/hypertensive state. Discrepancies were resolved by consensus. All MRAs were reviewed on dedicated workstations (Insignia Medical Systems, United Kingdom). The visualised V2 (portion in the vertebral columns), V3 (after exit from the C2 transverse foramen) and V4 (the intracranial portion beginning at the atlanto-occipital membrane and terminating at the basilar artery) segments were analysed. VAH was defined as a diameter < 2 mm uniformly throughout the vessel, and not if only a focal narrowing was presented suggestive of atherosclerotic steno-occlusive disease, as previously described (Fig. 2) [19]. CoW anatomy was classified as previously described [20]. Briefly, vessels that were visualized as continuous segments of at least 0.8 mm in diameter were considered present and those smaller than 0.8 mm in diameter were considered hypoplastic [20]. These predefined caliber thresholds facilitated direct comparison between 1.5 T and slightly higher resolution 3 T MRA datasets. Care was taken to distinguish the posterior communicating arteries from the anterior choroidal arteries by cross-referencing MPR images. The communication of the posterior communicating artery with the posterior cerebral artery was confirmed for all posterior communicating arteries identified. The posterior aspect of each CoW was assessed for morphology and classified as previously described [20]. Incomplete posterior CoW was defined as either unilateral or bilateral hypoplastic or absent posterior communicating arteries or unilateral or bilateral hypoplastic or absent pre-communicating segment of the posterior cerebral artery or a combination thereof (Fig. 3).

### Statistical analysis

Statistical analysis was performed using SPSSv.21 (Statistical Package for the Social Sciences (SSPS), International Business Machines, Inc., Armonk, New York, USA). An overall sample size of 49 provides a power of > 80% to find a 27% difference (estimated from a prior study [12]) in the prevalence of VAH between the two groups, with a two-sided type one error of 0.05. All data analysis was blinded. Normality was determined by the Shapiro-Wilks test. Differences between: 1) controls and CoA subjects and 2) CoA subjects with VAH + ipCoW and CoA subjects without VAH + ipCoW were assessed by unpaired Students t-tests, independent samples Mann-Whitney U tests or Fisher’s exact tests as appropriate. Binary logistic regression analysis was performed to determine differences, controlling for age, male gender and body mass index (BMI), in odds ratios for: 1) the presence of VAH + ipCoW in CoA compared to normotensives and 2) the presence of a diagnosis of hypertension in subjects with VAH + ipCoW compared to those without. Differences in prevalence of VAH + ipCoW between CoA hypertensive and non-hypertensive subgroups was assessed with a Fisher’s exact test. Univariate and multivariate regression analysis was performed to assess for determinants of VAH + ipCoW in repaired CoA subjects. Where appropriate, data are reported as mean ± standard deviation, median with range, as a percentage or odds ratio with 95% confidence intervals. All statistical tests were two-tailed. Significance was set at *P* <  0.05.

## Results

The demographic data are described in Table 1. There were no significant differences between post-repaired CoA subjects and controls in sex (male: 61% vs 48%, *p* = 0.24) or body mass index (BMI) (25 ± 5 vs 24 ± 3 kg/m^2^, *p* = 0.23). Mean age of the post-repaired CoA cohort was significantly lower than controls (34 ± 14 vs 42 ± 14 years, *p* = 0.002). Subsequent analyses were corrected for baseline differences in the covariates of age, male sex and BMI. Amongst the repaired CoA cohort, non-stent treatment of coarctation was performed in the vast majority of patients 94% (119/127), consisting of end to end anastomosis 38% (48/127), subclavian flap 15% (19/127), dacron patch 11% (14/127) and balloon angioplasty 6% (8/127) and 24% (30/127) non-stent repair with incomplete surgical history. No post-CoA repair subjects had clinical or imaging evidence of significant restenosis. No patients had renal artery stenosis.

**Table 1:** Baseline demographics and cerebrovascular variant prevalence

	Healthy Controls (*n* = 33)	Coarctation (*n* = 127)	*p*-value
Demographic data			
Age (years)	42 ± 14	34 ± 14	= 0.002
Male gender n [%]	16 [48]	77 [61]	= 0.24
BMI (kg/m^2^)	24 ± 3	25 ± 5	= 0.23
Office SBP (mmHg)	124 ± 10	138 ± 19	< 0.0001
Office DBP (mmHg)	76 ± 8	76 ± 11	=0.94
Vertebral artery and Circle of Willis data			
VAH n [%]	8 [24]	57 [45]	= 0.046
ipCoW n [%]	19 [58]	79 [62]	= 0.69
VAH + incomplete pCoW n [%]	3 [9]	46 [36]	= 0.003

*BMI* body mass index, *DBP* diastolic blood pressure, *ipCoW* incomplete Circle of Willis, *CoW SBP* systolic blood pressure, *SBP* systolic blood pressure, *VAH* vertebral artery hypoplasia

### VAH and ipCoW occurs more frequently in repaired CoA than controls

We addressed the question: does VAH and ipCoW occur more frequently in repaired CoA subjects, a requisite if the “selfish brain” hypothesis is implicated in the development of hypertension following repaired CoA. Odds ratios from multivariate logistic regression analysis, controlling for age, male gender and BMI, showed that patients with repaired CoA (*n* = 127) were 5.8 times more likely to have VAH + ipCoW than controls (*n* = 33)(β: 5.795, 95th CI: 1.614–20.812, *p* = 0.007) but age (β: 1.013, 95th CI: 0.985–1.041, *p* = 0.37), male gender (β: 1.429, 95th CI: 0.681–2.999, *p* = 0.35) and BMI (β: 1.038, 95th CI: 0.966–1.115, *p* = 0.31) were not significant predictors of VAH and ipCoW.

### VAH and iCoW is a predictor of hypertension in repaired CoA

Next, we sought to answer the question: does VAH + ipCoW predict hypertension following CoA repair? Prevalence of VAH and ipCoW in coarctation with hypertension (*n* = 64) was significantly higher than controls (*n* = 33) (44% vs 9%, *p* <  0.0001, Fisher’s exact test). Additionally, the prevalence of VAH and ipCoW in coarctation without hypertension or anti-hypertensive medication (*n* = 63) was significantly higher than controls (*n* = 33) (29% vs 9%, *p* = 0.037, Fisher’s exact test). There were no differences in percentage restenosis at the site of CoA repair (17 ± 27% vs 16 ± 19%, *p* = 0.74) or degree of arch hypoplasia (arch hypoplasia index: 0.79 ± 0.20 vs 0.76 ± 0.17, *p* = 0.32) between repaired CoA cohorts with or without hypertension. However, there was only a trend towards higher prevalence of hypertension in repaired CoA with VAH + ipCoW compared to those without (61% vs 44%, *p* = 0.097) but repaired CoA subjects with VAH + ipCoW had higher SBP (145 ± 20 vs 134 ± 18 mmHg, *p* = 0.003) despite more having anti-hypertensive medications prescribed (54% vs 33%, *p* = 0.025). Furthermore, repaired CoA subjects with VAH + ipCoW and hypertension were older than those without hypertension (38 ± 11 vs 30 ± 12 years, *p* = 0.035).

In multivariate logistic regression analysis, correcting for age, male gender and BMI, VAH + ipCoW was a significant independent predictor of a diagnosis of hypertension; where present, this increased the odds of a diagnosis of hypertension independently by 2.5 times (β: 2.473, 95th CI: 1.173–5.212, *p* = 0.017).

### VAH ipCoW is associated with higher BP and uncontrolled hypertension

Not all subjects with repaired CoA have VAH + ipCoW. Subgroup analysis comparing repaired CoA subjects without VAH + ipCoW (*n* = 81) and subjects with VAH + ipCoW (*n* = 46) was performed to determine if these cerebrovascular variants were associated with higher BP and uncontrolled hypertension. The subgroup analysis is presented in Table 2. Odds ratios from binary logistic regression showed that when VAH + ipCoW were present, subjects were 3.3 times more likely to have treated uncontrolled hypertension (β: 3.286, 95th CI: 1.005–10.743, *p* = 0.049).

**Table 2:** Subgroup analysis of repaired CoA subject with and without VAH + ipCoW

	Repaired coarctation patients		*p*-value
	No VAH + ipCoW (*n* = 81)	VAH + ipCoW (*n* = 46)	
Demographic data			
Age (years)	33 ± 14	35 ± 12	= 0.32
Male gender n [%]	46 [57]	31 [67]	= 0.26
BMI (kg/m^2^)	25 ± 5	26 ± 5	= 0.13
Blood pressure data			
Office SBP (mmHg)	134 ± 18	145 ± 20	< 0.003
Office DBP (mmHg)	75 ± 10	77 ± 11	= 0.17
Uncontrolled HTN^a^ n [%]	5 [6]	8 [18]	= 0.064
On anti-HTN Rx n [%]	27 [33]	25 [54]	= 0.025
ACEi /ARB n [%]	41 [50]	29 [64]	= 0.31
CCB n [%]	16 [20]	12 [26]	= 0.74
Beta-blocker n [%]	36 [44]	19 [42]	= 0.99
Other congenital heart defects			
Bicuspid aortic valve n [%]	66 [81]	28 [61]	= 0.14
Ventriculoseptal defect	13 [16]	4 [9]	= 0.30
Coarctation repair data^b^			
Age at repair (years)	5 (0–39)	7 (0–59)	= 0.41
End to End repair n [%]	29 [47]	19 [51]	= 0.68
Subclavian flap n [%]	14 [23]	6 [16]	= 0.61
Patch repair n [%]	8 [13]	7 [19]	= 0.56
Angioplasty^c^ n [%]	11 [18]	5 [14]	= 0.78
Recoarctation n [%]	43 [52]	19 [41]	= 0.35

*ACEi* angiotensin converting enzyme inhibitor, *ARB* angiotensin receptor blocker, *CCB* calcium channel blocker, *HTN* hypertension

^a^ Uncontrolled hypertension definition: office BP > 140/90 mmHg despite at least 2 anti-hypertensive medications

^b^ Repair data = median (range), total *n* = 99, no VAH + iCoW *n* = 62, VAH + iCoW *n* = 37

^c^ Angioplasty is pooled balloon and stent angioplasty subgroups

Not all subjects with repaired CoA and VAH + ipCoW had hypertension. Subgroup analysis demonstrated that subjects with repaired CoA, VAH + ipCoW and hypertension (*n* = 28) were significantly older than subjects with repaired CoA, VAH + ipCoW without hypertension (*n* = 18) (38 ± 11 vs 30 ± 12 years, *p* = 0.035, Student’s t-test) but there were no significant differences in BMI (26 ± 5 vs 26 ± 7 kg/m^2^, *p* = 0.99, Student’s t-test) or sex (75% (21/28) male vs 56% (10/18) male, *p* = 0.21, Fisher’s Exact test). There were no significant differences in prevalence of repair types between repaired CoA with VAH + ipCoW and hypertension compared to those without hypertension. In addition, Amongst subjects with repaired coarctation but without VAH + ipCoW, those with hypertension were older than those still normotensive (37 ± 15 vs 29 ± 11 years, *p* = 0.013, Student’s t-test).

### Determinants of VAH and ipCoW in repaired CoA

**Fig. 4:**
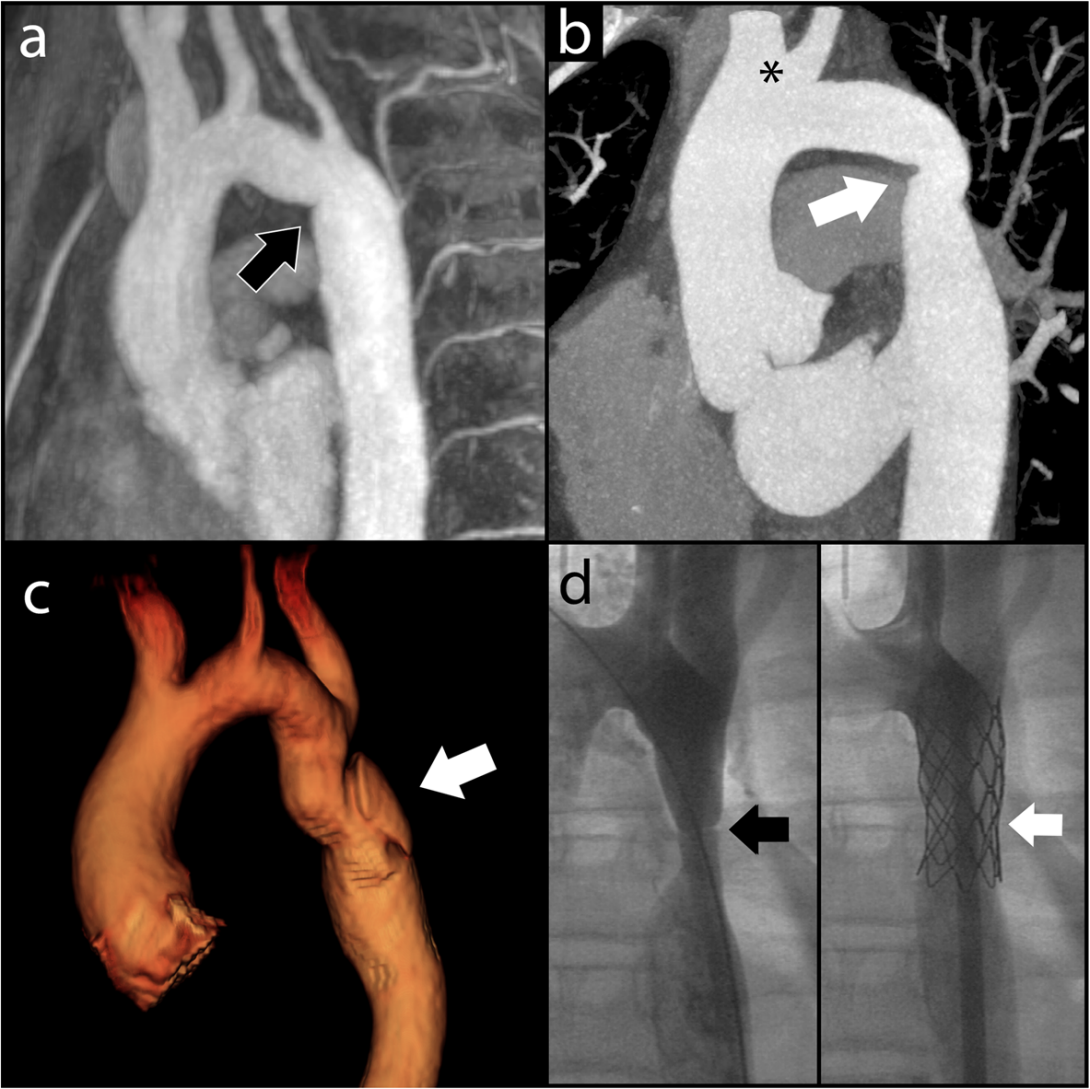
Examples of CoA repair. **a** Oblique sagittal maximum intensity projection reconstruction of MRA performed for a patient who underwent end-to-end anastomotic CoA repair. A mild fold is demonstrated at the CoA repair site at the aortic isthmus (arrow). **b** Oblique sagittal maximum intensity projection reconstruction CT angiogram for patient with subclavian flap CoA repair. There is absence of the proximal left subclavian artery with mild narrowing at the site of coarctation repair in the distal arch (arrow). Note normal variant conjoint origin of the right brachiocephalic and left common carotid artery (asterisk). **c** 3D reconstruction MRA in a patient who underwent patch repair of significant CoA. The white arrow indicates a pseudoaneurysm in the proximal descending aorta, which developed at the site of repair. **d** Fluoroscopic images of CoA stent procedure. Left panel shows CoA in the proximal descending aorta (black arrow). Right panel shows successful stent implantation with improved patency of the proximal descending aorta (white arrow)

Finally, we sought to determine whether the presence of VAH and ipCoW in repaired CoA was related to the either the age at time of repair or the type of CoA repair. In the subgroup of patients with adequate clinical history of time and type of repair (*n* = 100), age at time of repair was not a predictor of VAH and ipCoW in univariate or multivariate analysis, accounting for gender and BMI (Table 3). None of the types of repair (end to end anastomosis, subclavian flap repair, patch repair, balloon / stent angioplasty) (Fig. 4) were predictors of VAH + ipCoW in univariate analysis (Table 3).

**Table 3:** Univariate and multivariate logistic regression for determinants of VAH + ipCoW in repaired CoA

	Univariate analysis		Multivariate analysis	
	OR (95% CI)	*P*-value	OR (95% CI)	*P*-value
Age at time of repair (years)	1.03 (0.99–1.07)	= 0.08	1.03 (0.99–1.07)	= 0.12
Male gender	2.15 (0.91–5.08)	= 0.08	2.46 (0.98–6.18)	= 0.06
BMI (kg/m^2^)	1.06 (0.98–1.14)	= 0.15	1.06 (0.98–1.15)	= 0.15
End to End repair	1.11 (0.49–2.50)	= 0.80	…	
Subclavian flap repair	0.68 (0.24–1.95)	= 0.47	…	
Patch repair	1.60 (0.53–4.86)	= 0.40	…	
Balloon / Stent angioplasty	0.74 (0.24–2.32)	= 0.60	…	
Recoarctation	1.34 (0.66–2.70)	= 0.42	…	

*OR* odds ratio, *CI* confidence interval

## Discussion

For the first time, we investigated the “selfish brain” hypothesis in hypertension following surgical CoA repair. Our novel findings are: 1) there is a higher prevalence of VAH + ipCoW in repaired CoA patients than controls, 2) VAH + ipCoW is an independent predictor of hypertension after controlling for age, gender and BMI, 3) repaired CoA subjects with VAH + ipCoW are more likely to have higher BP and uncontrolled hypertension than those without and 4) neither the age at time of repair nor any specific repair type were significant predictors of VAH + ipCoW. Together, these findings suggest that VAH + ipCoW in repaired CoA subjects may be congenital or acquired, independent of the timing and type of repair and could account for the development of hypertension or its persistence, following CoA repair.

SNA is elevated in most hypertensive humans [21–23] and in patients with a history of CoA and hypertension [10]. However, the driver behind the elevated SNA is not clear. Both work in the spontaneously hypertensive rat model [24] and post-mortem humans studies [25] have suggested that brain blood flow is crucial in determining SNA and therefore systemic arterial pressure. At post-mortem, Dickinson and Thomason demonstrated that vertebral arteries in patients with ante-mortem hypertension were narrower than those who were normotensive ante-mortem and that vertebral artery resistance correlated positively with ante-mortem blood pressure [25]. This paved the way for the development of “the selfish brain hypothesis” of hypertension, which proposes that vertebral artery narrowing with resultant brainstem hypoperfusion results in neurogenic-mediated increases in SNA in an attempt to increase systemic pressure to compensate for the decreased brain blood flow [24] [26]. This is supported by rat data that demonstrate that vertebrobasilar artery hypertrophy occurs prior to the development of hypertension and that brainstem ischaemia from bilateral vertebral artery clamping results in significantly increased SNA in pre-hypertensive spontaneously hypertensive rats compared to age-match normotensive rats [24]. Moreover, the brainstem of the spontaneously hypertensive rat is hypoxic compared to the normotensive rat at the same level of blood oxygenation and blood pressure, both at hypertensive levels and markedly so when blood pressure falls [27].

Recently, our group provided in-vivo evidence for the “selfish brain” hypothesis by demonstrating that humans with hypertension had a higher prevalence of VAH + ipCoW, which was coupled with elevated cerebrovascular resistance and diminished cerebral blood flow [12]. Importantly, cerebral vascular resistance was increased prior to the development of hypertension and elevated SNA was also increased in untreated borderline hypertensive subjects, suggesting a causal link [12]. The findings of the current study provide the first evidence to support a similar pathophysiological process occurring in, at least some, subjects following CoA repair. All patients with repaired CoA undergo assessment of their blood pressure on an annual basis, but it may be that those with VAH + ipCoW need to have more vigilant assessment with more frequent ambulatory blood pressure monitoring, for example.

Our findings raise several important questions. Firstly, when does VAH + ipCoW occur in subjects with CoA? There are at least two possibilities: 1) VAH + ipCoW occurs as a result of CoA, in an attempt to protect the brain from elevated perfusion pressures that may occur in the face of the central aortic obstruction or 2) VAH + ipCoW occurs in conjunction with CoA as a manifestation of a more widespread congenital vascular abnormality. If the former is the case, the severity and duration of the aortic obstruction would likely be important factors in the development of the cerebrovascular variants but we did not demonstrate age at time of repair to be a significant predictor of VAH + ipCoW. The fact that subjects with CoA manifest pathological adjustment of autonomic cardiovascular homeostasis as early as the neonatal period before surgical repair [10] supports the latter developmental hypothesis. Future longitudinal follow-up studies of subjects with CoA, starting as early as in-utero, with genotyping and paired serial MRI assessments of cerebrovascular anatomy / resistance and assessment of SNA will help further clarify the answer to this question. Interestingly, amongst repaired coarctation subjects without VAH + ipCoW, those who were hypertensive were old than those who were normotensive. It is possible that a significant proportion of patients with repaired coarctation are still destined to become hypertensive and the presence of VAH + ipCoW may accelerate this process. Longitudinal studies will also help address this hypothesis.

Regardless of the etiology of the VAH + ipCoW, it is interesting to postulate the impact of the current management of relieving the central aortic obstruction on the cerebral perfusion in individuals with VAH + ipCoW. The anticipated reduction in central aortic pressure following treatment for CoA could potentially aggravate cerebral perfusion in individuals with at risk vasculature. Indeed, in previous work, treatment controlled hypertensive participants had significantly lower cerebral perfusion than normotensive controls [12] and cerebral blood flow has been demonstrated to be lower in patients with CoA [28]. If this were the case, CoA repair may actually predispose certain subjects to increased neurogenic-mediated SNA. Supporting this notion, increased SNA has been documented in patients after CoA repair [29] and so-called paradoxical hypertension after treatment of CoA is well-recognized [30]. Determining the prevalence of VAH + ipCoW pre and post intervention, as well as assessing for change in cerebrovascular resistance and perfusion and SNA before and after treatment, will be important to determine the treatment effect on cerebrovascular function.

An important question that remains unanswered is: why do some patients with repaired CoA and VAH + ipCoW develop hypertension and others do not? The current study provides one possible explanation; the group with VAH + ipCoW without hypertension were significantly younger than the group with VAH + ipCoW and hypertension. It is possible that the subjects with the cerebrovascular variants have yet to develop hypertension in this single time-point cross-sectional study. Cerebral autoregulation appears to be impaired in CoA patients [28], which supports this notion. Longitudinal follow-up for this subgroup in particular will be important to document the incidence of hypertension. It is also important to realize that not all cases of hypertension following CoA repair can be attributed to VAH + ipCoW. There are many other potential causes including endothelial dysfunction [31] and arterial stiffness [32], either inherent or due to the presence of a stent as well as suboptimal haemodynamic repair. Additionally, there are other potential considerations that could specifically account for elevated SNA and hypertension beyond VAH + ipCoW, such as renovascular causes of hypertension.

## Limitations

This was retrospective analysis of a prospective database of repaired CoA subjects surviving to adulthood to be seen in a tertiary adult congenital heart disease clinic in the South West of England. This will unavoidably have introduced an element of survival bias into the study sample. This also constrained our analysis to anatomical assessment of VAH + ipCoW. However, our previous work has described the pathophysiological mechanisms associated with this finding in subjects with and without hypertension, which we assume to be similar in the current cohort [12]. In particular, it was demonstrated that the contralateral vertebral artery does not compensate for the hypoplastic artery in terms of cerebral perfusion [12].

The lack of ambulatory blood pressure data in all subjects is a limitation.

No large differences in surgical repair type were found. However, the absolute numbers in these subgroups is small. Future study is warranted in a larger cohort to detect smaller differences between surgical repair types, particularly since young children undergoing subclavian flap repair have previously been demonstrate to have higher blood pressure and stiffer upper limb arteries compared with matched children undergoing end-to-end anastomosis [33].

## Conclusion

VAH + ipCoW predicts hypertension and difficult to treat hypertension in repaired CoA. It is unrelated to age at time of repair or repair type. CoA may be a marker of wider congenital cerebrovascular problems. Understanding the “selfish brain” in CoA repair may help in identifying those patients at highest risk of developing hypertension, although further research is needed to guide an effective treatment strategy.

## Data Availability

The datasets used and/or analysed during the current study are available from the corresponding author on reasonable request.

## Abbreviations

BMI: Body mass index
CoA: Coarctation of the aorta
CVR: Cerebrovascular resistance
DBP: Diastolic blood pressure
ipCoW: Incomplete posterior circle of Willis
MPR: Multiplanar reformatted
MRA: Magnetic resonance angiography
SBP: Systolic blood pressure
SNA: Sympathetic nerve activity
VAH: Vertebral artery hypoplasia

## References

[1] Reller MD, Strickland MJ, Riehle-Colarusso T, Mahle WT, Correa A (2008). Prevalence of congenital heart defects in metropolitan Atlanta, 1998-2005. J Pediatr.

[2] Hoffman JIE, Kaplan S (2002). The incidence of congenital heart disease. J Am Coll Cardiol.

[3] Maron BJ, Humphries JO, Rowe RD, Mellits ED (1973). Prognosis of surgically corrected coarctation of the aorta. A 20-year postoperative appraisal. Circulation.

[4] Cohen M, Fuster V, Steele PM, Driscoll D, McGoon DC (1989). Coarctation of the aorta. Long-term follow-up and prediction of outcome after surgical correction. Circulation.

[5] Koller M, Rothlin M, Senning A (1987). Coarctation of the aorta: review of 362 operated patients. Long-term follow-up and assessment of prognostic variables. Eur Heart J.

[6] Toro-Salazar OH, Steinberger J, Thomas W, Rocchini AP, Carpenter B, Moller JH (2002). Long-term follow-up of patients after coarctation of the aorta repair. Am J Cardiol.

[7] O’Sullivan JJ, Derrick G, Darnell R (2002). Prevalence of hypertension in children after early repair of coarctation of the aorta: a cohort study using casual and 24 hour blood pressure measurement. Heart.

[8] Freed MD, Rocchini A, Rosenthal A, Nadas AS, Castaneda AR (1979). Exercise-induced hypertension after surgical repair of coarctation of the aorta. Am J Cardiol.

[9] Ong CM, Canter CE, Gutierrez FR, Sekarski DR, Goldring DR (1992). Increased stiffness and persistent narrowing of the aorta after successful repair of coarctation of the aorta: relationship to left ventricular mass and blood pressure at rest and with exercise. Am Heart J.

[10] Polson Jaimie W., McCallion Naomi, Waki Hidefumi, Thorne Gareth, Tooley Mark A., Paton Julian F.R., Wolf Andrew R. (2006). Evidence for Cardiovascular Autonomic Dysfunction in Neonates With Coarctation of the Aorta. Circulation.

[11] Bergdahl L, Björk VO, Jonasson R (1983). Surgical correction of coarctation of the aorta. Influence of age on late results. J Thorac Cardiovasc Surg.

[12] Warnert EAH, Rodrigues JCL, Burchell AE et al. Is High Blood Pressure Self-Protection for the Brain? Circ Res. 2016;119(12):e140–e151. 10.1161/CIRCRESAHA.116.309493 27672161

[13] Mancia G, Fagard R, Narkiewicz et al. 2013 ESH/ESC guidelines for the management of arterial hypertension: the Task Force for the Management of Arterial Hypertension of the European Society of Hypertension (ESH) and of the European Society of Cardiology (ESC). Eur Heart J. 2013;34(28):2159–219. 10.1093/eurheartj/eht151 23771844

[14] Curtis SL, Bradley M, Wilde P et al. Results of Screening for Intracranial Aneurysms in Patients with Coarctation of the Aorta. 2012:1182–186. 10.3174/ajnr.A2915 PMC8013223 22322607

[15] Stern Heiko C., Locher Dietrich, Wallnöfer Klaus, Weber Fritz, Scheid Karl F., Emmrich Peter, Bühlmeyer Konrad (1991). Noninvasive assessment of coarctation of the aorta: Comparative measurements by two-dimensional echocardiography, magnetic resonance, and angiography. Pediatric Cardiology.

[16] Therrien J, Thorne SA, Wright A, Kilner PJ, Somerville J (2000). Repaired coarctation: a “cost-effective” approach to identify complications in adults. J Am Coll Cardiol.

[17] Quennelle S, Powell AJ, Geva T, Prakash A (2015). Persistent aortic arch hypoplasia after Coarctation treatment is associated with late systemic hypertension. J Am Heart Assoc.

[18] Safian RD, Textor SC (2001). Renal-artery stenosis. N Engl J Med.

[19] Park J-H, Kim J-M, Roh J-K (2007). Hypoplastic vertebral artery: frequency and associations with ischaemic stroke territory. J Neurol Neurosurg Psychiatry.

[20] Krabbe-Hartkamp M J, van der Grond J, de Leeuw F E, de Groot J C, Algra A, Hillen B, Breteler M M, Mali W P (1998). Circle of Willis: morphologic variation on three-dimensional time-of-flight MR angiograms. Radiology.

[21] Schlaich Markus P., Lambert Elisabeth, Kaye David M., Krozowski Zygmunt, Campbell Duncan J., Lambert Gavin, Hastings Jacqui, Aggarwal Anuradha, Esler Murray D. (2004). Sympathetic Augmentation in Hypertension. Hypertension.

[22] Wallin BG, Delius W, Hagbarth KE (1973). Comparison of sympathetic nerve activity in normotensive and hypertensive subjects. Circ Res.

[23] Grassi G, Cattaneo BM, Seravalle G, Lanfranchi A, Mancia G (1998). Baroreflex control of sympathetic nerve activity in essential and secondary hypertension. Hypertens (Dallas, Tex 1979).

[24] Cates MJ, Steed PW, Abdala APL, Langton PD, Paton JFR (2011). Elevated vertebrobasilar artery resistance in neonatal spontaneously hypertensive rats. J Appl Physiol.

[25] Dickinson C.J., Thomson A.D. (1959). VERTEBRAL AND INTERNAL CAROTID ARTERIES IN RELATION TO HYPERTENSION AND CEREBROVASCULAR DISEASE. The Lancet.

[26] Cates MJ, Dickinson CJ, Hart ECJ, Paton JFR (2012). Neurogenic hypertension and elevated vertebrobasilar arterial resistance: is there a causative link?. Curr Hypertens Rep.

[27] Marina Nephtali, Ang Richard, Machhada Asif, Kasymov Vitaliy, Karagiannis Anastassios, Hosford Patrick S., Mosienko Valentina, Teschemacher Anja G., Vihko Pirkko, Paton Julian F. R., Kasparov Sergey, Gourine Alexander V. (2015). Brainstem Hypoxia Contributes to the Development of Hypertension in the Spontaneously Hypertensive Rat. Hypertension.

[28] Wong Rachel, Ahmad Waheed, Davies Allan, Spratt Neil, Boyle Andrew, Levi Christopher, Howe Peter, Collins Nicholas (2017). Assessment of cerebral blood flow in adult patients with aortic coarctation. Cardiology in the Young.

[29] Lee Melissa G.Y., Hemmes Robyn A., Mynard Jonathan, Lambert Elisabeth, Head Geoffrey A., Cheung Michael M.H., Konstantinov Igor E., Brizard Christian P., Lambert Gavin, d'Udekem Yves (2017). Elevated sympathetic activity, endothelial dysfunction, and late hypertension after repair of coarctation of the aorta. International Journal of Cardiology.

[30] Sealy WC (1990). Paradoxical hypertension after repair of coarctation of the aorta: a review of its causes. Ann Thorac Surg.

[31] Kenny D, Polson JW, Martin RP, Paton JF, Wolf AR (2011). Hypertension and coarctation of the aorta: an inevitable consequence of developmental pathophysiology. Hypertens Res.

[32] Kenny Damien, Polson Jaimie W., Martin Robin P., Caputo Massimo, Wilson Dirk G., Cockcroft John R., Paton Julian F.R., Wolf Andrew R. (2011). Relationship of aortic pulse wave velocity and baroreceptor reflex sensitivity to blood pressure control in patients with repaired coarctation of the aorta. American Heart Journal.

[33] Kenny Damien, Polson Jaimie W., Martin Robin P., Wilson Dirk G., Caputo Massimo, Cockcroft John R., Paton Julian F.R., Wolf Andrew R. (2010). Surgical Approach for Aortic Coarctation Influences Arterial Compliance and Blood Pressure Control. The Annals of Thoracic Surgery.

